# Exacerbation of Fatality Rates Induced by Poor Air Quality Due to Open-Air Mass Funeral Pyre Cremation during the Second Wave of COVID-19

**DOI:** 10.3390/toxics10060306

**Published:** 2022-06-06

**Authors:** M. G. Manoj, M. K. Satheesh Kumar, K. T. Valsaraj, Soumya K. Vijayan, T. Nishanth

**Affiliations:** 1 Advanced Centre for Atmospheric Radar Research, Cochin University of Science and Technology, Cochin 682022, India; 2 Department of Atomic and Molecular Physics, Manipal Academy of Higher Education, Manipal 576104, India; drmksatheesh@gmail.com; 3 Cain Department of Chemical Engineering, Louisiana State University, Baton Rouge, LA 70803, USA; valsaraj@lsu.edu; 4 College of Pharmaceutical Sciences, Government Medical College, Kannur 670503, India; skv4biotech@gmail.com; 5 Department of Physics, Sree Krishna College, Guruvayur 680102, Kerala, India; nisthu.t@gmail.com

**Keywords:** COVID-19, air pollution, mass cremation, funeral pyre, surface ozone

## Abstract

This study investigates the air pollution-induced mortality rate during the second wave of COVID-19, which claimed several thousand lives in the capital city of India, New Delhi, even during a lockdown period. Delhi is a hotspot of unhealthy air quality. During the second wave of COVID-19 in 2021, surface ozone levels were observed to be higher, which had a direct impact on lung function, thereby making people more susceptible to COVID-19. The correlation coefficient between surface ozone concentration and mortality has been observed to be 0.74 at a 95% confidence level. This work focuses on the plausible impact and feedback of poor air quality induced by the burning of open-air funeral pyres due to the increased COVID-19 mortality rate in New Delhi, estimated by using an epidemiological model (AirQ+) of the World Health Organization. The mortality rate estimated quantitatively with the aid of AirQ+ is 1.27 excess deaths per 100,000 population due to surface ozone from pyre burning. The findings suggest transformational system goals before the resurgence of a subsequent wave.

## 1. Introduction

**Figure 1:**
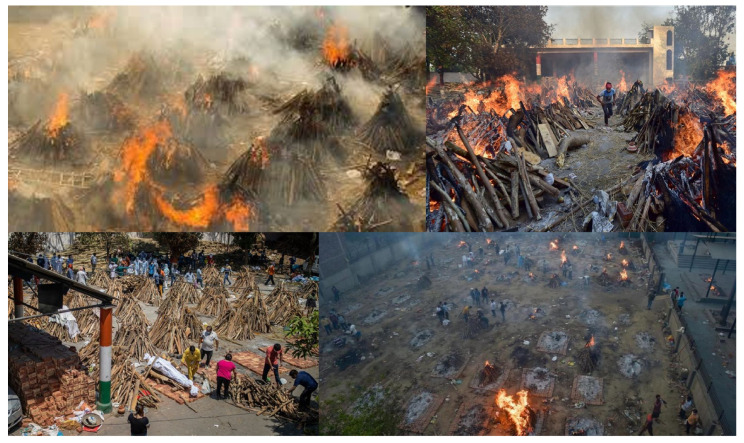
Massive open-air pyre for cremation of COVID-19 victims at various crematoriums in New Delhi during March through May in 2021, reported in the media. (Images courtesy of (**top left**) https://www.newindianexpress.com, accessed on 12 May 2022 (**top right**) www.oneindia.com, accessed on 12 May 2022 (**bottom left**) https://www.outlookindia.com, accessed on 12 May 2022 (**bottom right**) https://in.news.yahoo.com, accessed on 12 May 2022).

The resurgence of COVID-19 patients in New Delhi, the National Capital Region (NCR) of India, during the second wave was witnessed in February 2021 and reached its maximum phase in April 2021. The combination of a large asymptomatic population and the presence of more infectious variants of the virus during the second wave, which is much steeper than the first wave that peaked in September 2020, led to the continued transmission of the virus even among those who remained indoors (https://science.thewire.in/health/sars-cov-2-variants-b117-b1617-india-second-wave-uncertain-future/, accessed on 12 May 2022). This created an intense second wave of infection and its alarming spread seriously affected life in the city of New Delhi. Further, the acute shortage of oxygen cylinders and beds in intensive care units in hospitals created a pandemic situation that placed extraordinary pressure on families to streamline the oxygen supply to dying patients. Thus, a crisis emerged in the NCR and surrounding regions due to the unprecedented and escalating spread of the coronavirus on an outsized scale. As a result, patients in critical condition were unable to receive hospital admissions and most patients were advised to stay at home, which contributed to the increased spread of the virus. Likewise, the surge of the epidemic simultaneously in multiple locations, in turn, produced a rise in cases. Subsequently, the increased Test Positivity Rate (TPR), peaking at 32.82%, resulted in a high mortality rate in the region, in which a large number of corpses were being transported to the crematoriums in and around Delhi. To contain the spread of the disease, and in the context of the record rise in COVID-19 cases, the Delhi Government announced a complete one-week lockdown in the capital, starting from the night of 19 April 2021 and further extended to 31 May 2021 to impose social distancing after the death toll started to rise. The mounting death toll led to an overflow of victims waiting for cremation at existing crematoriums with limited openings, and even open grounds were converted into temporary crematoriums for mass cremation (Figure 1; an article also appeared in a local newspaper describing the situation wherein residents were unable to tolerate the smoke being emitted from the pyres in another Indian city, Hyderabad, which is provided in Figure S1).

A recent study by Patel et al. [1] points to the fact that, despite the lockdown, the reduction in certain primary pollutant concentrations was, rather, only marginal compared to the baseline values, and there was a dominance of secondary aerosol pollution. The surface ozone was shown to be on the increasing side due to the decline in volatile organic compound (VOC) levels during most of the lockdown phases. Despite the reduction in local and regional emissions due to public transportation, construction activities, and agricultural burning, the enhancement in certain pollutant levels requires the consideration of extraneous factors in the poor air quality experienced across Delhi. Another study by Antil et al. [2] reports that while the levels of many pollutants, such as PM_10_, PM_2.5_, SO_2_, NO_x_, etc., have decreased in several areas in Delhi, the surface ozone level has increased consistently, which requires detailed investigation. This short paper explores the impact of massive open-air cremation on the air quality over Delhi during the peak phase of the second wave, and its plausible impact on the mortality rate through a feedback mechanism. Though the emissions resulting from funeral pyre burning comprise several particulate and trace gases, we focus more on the impact of surface ozone, a toxic gas present over the NCR during this period.

## 2. Materials and Methods

### 2.1. Data and Geographical Features of Delhi

COVID-19 infection and mortality rate data during the peak period of 16 March through 24 May 2021 were acquired from the official health bulletin released by the Delhi State Government (https://www.covid19india.org/, accessed on 12 May 2022). The air quality data over Delhi were accessed from the publicly available repository of the Central Pollution Control Board under the Ministry of Environment, Forests and Climate Change, Government of India (https://app.cpcbccr.com/AQI_India/, accessed on 12 May 2022).

Concentrations of various pollutants, such as particulate matter (PM_10_ and PM_2.5_), carbon monoxide (CO), NO_2_ and surface ozone (O_3_), were obtained during this period for the continuously available sites and then averaged to obtain a gross representation. Pearson’s correlation analysis was performed to examine the association between the mortality rate and each of the pollution components. Inferences were made based on the strength of correlation and on physical reasoning based on known atmospheric chemistry. New Delhi, the capital of India, is located in Northern India between the latitudes of 28°24′17″ and 28°53′00″ N and longitudes of 76°50′24″ and 77°20′37″ E, which spans an area of 1483 km^2^. It is a highly urbanized, landlocked city with an elevation of 216 m above sea level. The population of Delhi is 16.78 million and approximately 97.5 percent of the population lives in urban areas, while the remaining 2.5 percent resides in rural areas, as per the 2011 census. The rate of growth of the population is 21.2% as per the 2011 census, and the crude death rate is 3.3 per 1000 population. The density of the population in New Delhi is 4057 persons per sq. km in 2011 (Source: http://delhiplanning.nic.in/sites/default/files/chapt1.pdf, accessed on 12 May 2022).

### 2.2. AirQ+: A Software Tool for Health Risk Assessment of Air Pollution

The AirQ+ software tool was developed by the World Health Organization (WHO, Europe) and is used to quantify the health effects of exposure to air pollution, including estimates of life expectancy (https://www.euro.who.int/en/health-topics/environment-and-health/air-quality/activities/airq-software-tool-for-health-risk-assessment-of-air-pollution, accessed on 12 May 2022). It is primarily used to estimate both the short-term (based on risk estimates from time-series studies) and long-term (using life-tables approach and risk estimates from cohort studies) effects of air pollution. Moreover, it is possible to estimate how much of a particular health effect is attributable to the selected air pollutants. AirQ+ enables users to pre-load data sets for air quality, relative risks (RRs) for selected pollutant health end-point pairs, population and baseline rates of health outcomes. The main methodologies use evidence-based epidemiological cohort studies, showing a relationship between average long-term air pollution concentration levels and the mortality risks in the exposed population. A brief explanation of the health impact assessment of surface ozone using AirQ+ is provided below.

#### Short-Term Impact of Ozone

To assess the health impacts of ozone, the user has to provide the value of an indicator, *SOM*35 (sum of ozone means over 35 ppb), defined as follows:

(1) $$SOM35_{uncorrected}=\sum_{n=1}^{n}max\left\{0,C_{i}-35\;\mathrm{ppb}\right\}$$

Here, $C_{i}$ is the daily maximum 8 h mean concentration and the summation is made from day *i* = 1 to *n*, with $N_{valid}$ as the number of days with valid values [3], from which the relative risk (*RR*) is estimated as

(2) $$RR=exp\left[\beta \times \frac{SOM35_{uncorrected}}{N_{valid}}\right]$$

Here, the parameter β = 0.000299 for a central value of *RR* = 1.003. A central value of 1.003 for *RR* with the lower and upper confidence limits of 0.997 and 1.0045, respectively, is used for Delhi based on published results [4,5]. The calculations are performed using Equations (1) and (2), with β values being computed using standard table values for ranges of ozone concentrations. The population attribution fraction (*PAF*) estimates the proportion of cases that would be prevented if exposure were zero. In the event that the whole population is exposed,

(3) $$PAF=\left[\frac{RR-1}{RR}\right]$$

Attributable burden (*AB*), the fraction of all cases attributable to a specific pollutant, is given by the product of PAF and the burden of disease (*BoD*), defined as the total burden of a specific health outcome:

(4) AB=BoD×PAF

Finally, the health outcome (such as mortality) of a particular pollutant, e.g., surface ozone, is estimated as

(5) $$AB_{ozone}\left(mortality\right)=\sum_{all\;age\;group}AB_{ozone}\left(mortality,\;age\;group\right)$$

### 2.3. Chemistry of Surface Ozone

In general, the chemistry of ground-level ozone from its precursors is quite complex. Ozone is a secondary air pollutant formed by the photo-dissociation of NO_2_ by solar radiation and destroyed by titration with NO. However, these reactions are modified by the presence of peroxyl radicals produced from the organic compounds present in the VOCs. The hydroperoxyl radical (HO_2_) produced from the OH reacts with CO in the presence of ambient O_2_ and oxidizes NO to NO_2_ [6]. Thus, CO enhances O_3_ production through an increased concentration of NO_2_. Normally, the smoke contains relatively high levels of CO (a product of incomplete combustion) and particulate matter, along with a huge amount of CO_2_, which is a serious concern in terms of deteriorating air quality. The enhanced emission of CO and NO_2_ could be a favorable factor in boosting the production of surface ozone through well-established ozone chemistry. This is well supported by the observed positive correlation coefficients of ozone with CO and NO_2_ over the location of study. The ozone production from CO depends on the ambient concentrations of volatile organic compounds. At lower temperatures, the smoke emission contains relatively high levels of CO (a product of incomplete combustion), and more particulate matter along with a high amount of CO_2_, which is also a major concern in terms of deteriorating air quality. Ozone, being a strong oxidizing agent, can cause the muscles in the airways to constrict, trapping air in the alveoli. This leads to severe inflammation in the lungs, which often causes wheezing and dyspnoea, a serious concern for COVID-19 patients on a large scale. COVID-19 patients, when exposed to such a damaging environment with a shortage of oxygen, are highly vulnerable. Ozone production in Delhi under normal conditions (before lockdown) follows the NOx-saturated or VOC-limited regime because of the high NOx level [7]. However, we presume that the lockdown-induced atmosphere in Delhi exhibited complex chemistry, which was modified to VOC-NOx-CO to demonstrate the real scenario, and a detailed investigation is required to retrieve the complex chemistry. A detailed description of all possible health outcomes of various pollutants for different age groups and genders can be found from the documentation of AirQ+ (link provided in Section 2.2). The latest version (v.2.1.1) of AirQ+ is used in the present study.

## 3. Results and Discussion

### 3.1. Role of Air Pollution in the Spread of COVID-19

**Figure 2:**
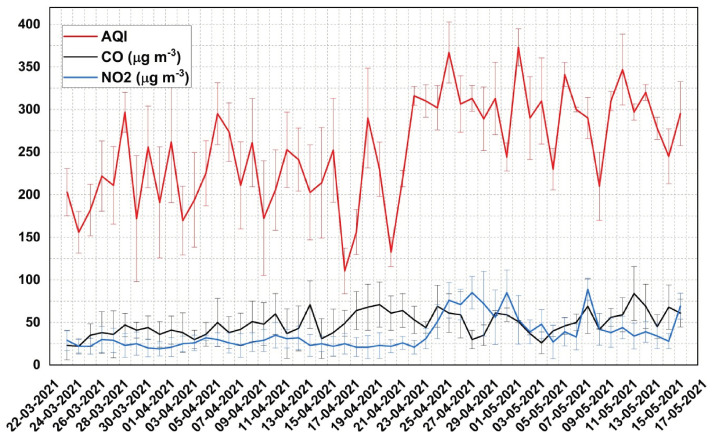
Daily variation in space–time-averaged values of air pollution. The variation in AQI, CO and NO${}_{2}$ during the peak of the second wave of the pandemic observed over Delhi on a daily basis.

Air pollution poses a major threat to human respiratory health and kills more than 7 million people annually all over the world, according to the World Health Organization [8]. This silent poisoning is facilitated by a variety of pollutant components, such as particulate matter (PM_10_, PM_2.5_), surface ozone (O_3_), oxides of nitrogen (NOx), carbon monoxide (CO), sulphur dioxide (SO_2_), etc. (source: https://www.health.nsw.gov.au/environment/air/Pages/common-air-pollutants.aspx, accessed on 12 May 2022). A multitude of studies have reported their health effects, such as upper and lower respiratory tract infections, irritation to the eyes, nose and throat, worsening asthma and the development of chronic bronchitis, cardiovascular diseases, premature childbirth and death, increased rate of disease progression and reductions in life expectancy (WHO, 2007). During this period of COVID-19 infections, it has been observed that the level of air pollution was much higher over the regions associated with the rapid spread of COVID-19. Analysis of the air quality index (AQI, Figure 2) reveals that all regions with a high mortality rate in Delhi witnessed an enhanced level of air pollution (frequently AQI > 175), which is classified as “unhealthy” and even “hazardous”, in the cold winter period and the subsequent months in the period of the second wave. Both short-term and long-term exposure to such a polluted environment could worsen lung functionality and even affect the lower respiratory tract, which carries an increased risk of COVID-19 infection for all age groups.

### 3.2. Emission from Burning Funeral Pyres

Open-air funeral pyre cremation is a traditional practice in South Asia, especially in India and Nepal, with more than 7 million human bodies burned every year using this method [9]. The authors adopt published results mostly from a robust study conducted by Chakrabarty et al. (2013) and references therein, for exploring the emission from burning pyres. As per this study, the average dry feedstock used per pyre is estimated to be 563 kg based on real-time measurements, and more than 4 Tg of raw material is consumed annually in India and Nepal in this practice. A typical pyre is 2 m in length and 1 m each in height and breadth (Figure 1). It is often constructed by using approximately 550 kg of wood, as well as a few kilograms of miscellaneous biological and synthetic materials, such as coconut shells, cow dung, rice grains, vermilion powder, camphor and clarified butter. The corpse is placed on top of the pyre and burning of the entire ensemble takes anywhere from 4 to 6 h, at rates ranging from 100 to 130 kg/hour. Quite often, the fire is a flaming–smoldering mixed phase and emits a brownish smoke. Smoldering combustion is a low-temperature oxidation process (<850 K) in the char layer that yields more CO and other incompletely oxidized pyrolysis products. The main components of the smoke from the solid feedstock samples used to build a pyre are carbon (both black and organic carbons), hydrogen, nitrogen, sulphur, oxygen and semi-volatile polycyclic aromatic hydrocarbons (PAH) and similar organic compounds [10]. The mean mass-weighted carbon mass fraction of the pyre feedstock and corpse has been measured to be 60%. This carbon mass often is converted to CO_2_, CO and particulate carbon, in order of abundance in the plume.

### 3.3. Observed Pollution and Its Relation to COVID-19 Mortality

The primary modes of transmission of COVID-19 are via droplets, contaminated hands and surfaces; however, airborne transmission was recently identified as the dominant route [11]. It is a well-established fact that polluted air aggravates chronic respiratory diseases and helps in the rapid transmission of the virus amongst the public [12]. Both short-term and long-term exposure to the polluted environment could worsen lung inflammation and affect even the lower respiratory tract, creating a dangerous environment conducive to COVID-19 transmission among all age groups. Smoke, which contains oxides of nitrogen and carbon, is classified as a prominent precursor of surface ozone. Ground-level ozone (O${}_{3}$), which is oxidative in nature, is produced by the photolysis of oxides of nitrogen by wavelengths less than 420 nm present in solar radiation in a rich environment of VOCs [7,13]. It can initiate Chronic Obstructive Pulmonary Disease (COPD) and asthma due to its strong oxidative capacity. Thus, the likelihood of COVID-19 infection is much higher in those who are already exposed to air pollutants capable of producing oxidative stress (Manoj et al., 2020). Delhi has experienced increased surface ozone, which is a secondary air pollutant produced from NOx-VOC (volatile organic compounds) chemistry in the presence of sunlight [7]. Generally, escalating numbers of vehicles and crop stubble burning are the primary sources of NOx, and VOCs produced by oil refineries and industries modulate surface ozone production. This ozone pollution is a severe issue that produces smog in Delhi, especially during winter, which deteriorates air quality and reduces visibility to a large extent [14,15]. The increase in the frequency of days with intense morning smog in Delhi has affected many aspects of human life, and it has been identified that surface ozone and particulate matter are the main air pollutants that create this haze, especially in the winter season. Thus, ozone has evolved as the primary air pollutant affecting the daily air quality index (AQI) after particulate matter on several days [16,17]. Prolonged inhalation of ozone, a strong oxidizing agent, could induce oxidative stress and cellular inflammation in the lungs, thereby damaging the inner lining of the lungs and thus increasing the severity of COVID-19 infection in patients of all ages.

**Figure 3:**
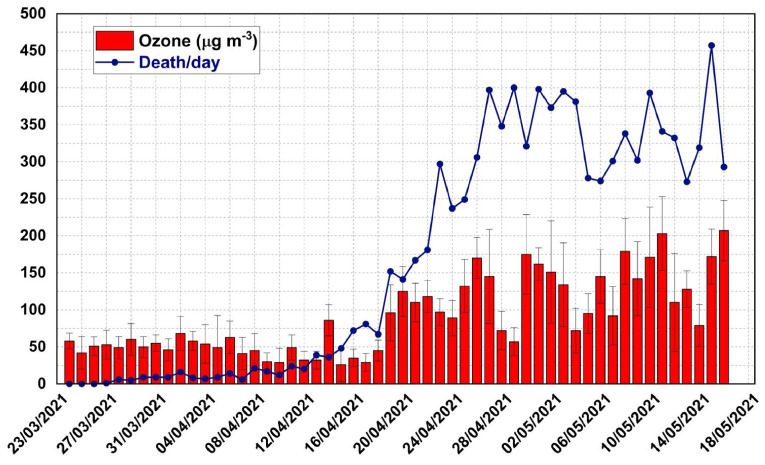
Co-variability of surface ozone and mortality. Daily variation in space–time-averaged surface ozone and death rate of COVID-19 patients during the peak of the second wave of the pandemic observed over Delhi.

After analyzing the air quality index (AQI) and its various components (O_3_, CO, NO_2_, PM_10_ and PM_2.5_) over Delhi during the second wave of COVID-19, we arrived at a meaningful inference on the pivotal role of air pollutants in worsening the critical condition due to COVID-19. A recent study [18] also reported an enhanced surface ozone level during the lockdown period in 2020 over Delhi. Figure 2 shows the variation in the AQI with the mortality rate during the peak phase of the second wave. A significant correlation (r = 0.7) at a 90% confidence level is found between the mean AQI and mortality rate. A poor association between mortality and particulate matter (PM_10_ and PM_2.5_) is obtained, probably due to the substantial reduction in the particulate matter as a result of the complete shutdown of almost all anthropogenic activities and industries due to lockdown. This is also evident from the weak and insignificant correlation of PM_10_ (r = 0.14) and PM_2.5_ (0.23) with the AQI, respectively. A higher correlation (0.57) is found between AQI and ozone, and a stronger relation between CO and ozone (r = 0.58) is also observed (Figure 2 and Figure 3). Similarly, ozone is related to NO_2_ through a significant correlation value (r = 0.57). The most striking feature in the present study is the significantly stronger correlation observed between surface ozone and mortality rate (r = 0.74; Figure 3) compared to other trace species considered here. This result is further confirmed by the presence of increased levels of surface ozone over Delhi, observed with satellite measurements. Analysis using satellite-derived tropospheric ozone (from AURA OMI data: https://disc.gsfc.nasa.gov/datasets/OMTO$_{3}$_003/summary, accessed on 12 May 2022) thus reveals enhanced surface ozone during the lockdown in 2021 (Figure S2). It is interesting to note that the tropospheric ozone increased in 2021 by almost 25% compared to its base value in 2020. The elevated ozone concentrations, observed towards the second leg of the lockdown phase, when the number of casualties was higher, indicates more ozone pollution resulting partly from pyre burning. The authors hypothesize that the massive funeral pyre cremation could have contributed significantly to the enhancement in surface ozone, through the chemical pathways discussed earlier, during this period, although an exact source apportionment study has not been carried out in real time. Thanks to the AirQ+ software tool, it is shown that a part of the excess mortality during the second wave can be attributed to the enhanced ozone levels due to pyre burning (refer to Section 3.3.1).

#### 3.3.1. Assessing the Impact of Pyre Burning on Excess Mortality

**Figure 4:**
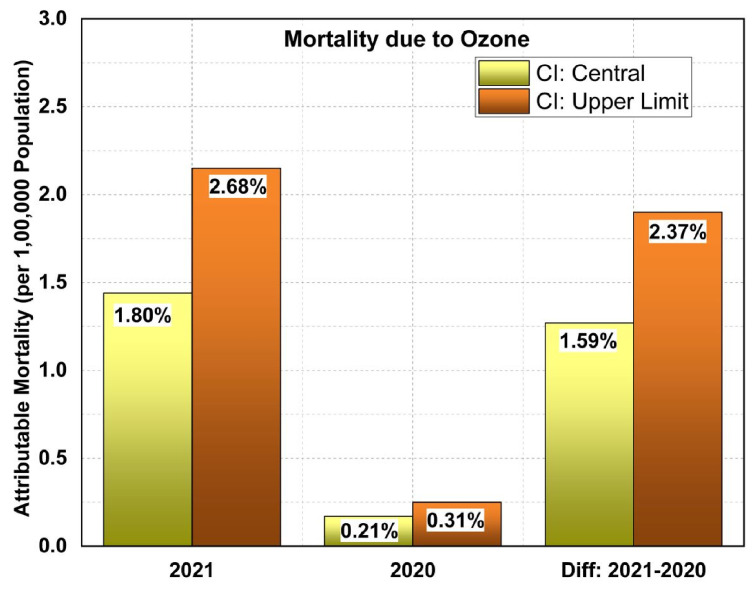
Attributable mortality (with confidence interval of central and upper limit) per 100,000 population due to surface ozone in the lockdown period in 2021, 2020, and the difference between them, estimated using the AirQ+ software tool (the percentage mortality is displayed on the bar graph).

To isolate the effects of pyre burning on excess ozone mortality, two lockdown periods, one in 2021 and the other in 2020, were considered and compared. The year 2021 witnessed the second wave of COVID-19, which claimed several thousands of lives compared to that in 2020, and the subsequent massive pyre burning reached a record level in 2021. Moreover, both the lockdowns were imposed during March–May of the respective year, implying nearly similar meteorological conditions. AirQ+ (v.2.1.1) was run with real-time data for each of the lockdown cases, and the attributable mortality due to ozone has been estimated. Figure 4 depicts the attributable mortality per 100,000 population for the cases during the years 2021 and 2020 and the difference between them. The central level of the confidence interval (CI) shows that ozone-attributed mortality was 1.80% (1.44 deaths per 1,00,000 population) in 2021 compared to 0.21% (0.17 deaths per lakh population) in 2020. The difference between these values amounts to 1.59% (1.27 death per 1 lakh population). Correspondingly, the upper level yields values of 2.68% (2.15 deaths), 0.31% (0.25) and 2.37% (1.90). Translating the central value of 1.59% (i.e., 1.27 deaths per 1,00,000 population) yields an excess mortality of 152 for the whole population over Delhi during 2021, resulting mostly from ozone pollution from pyre burning. Analogously, the upper limit (2.37%) sets an unprecedented record of 228 deaths solely due to funeral pyre pollution. Added to the previously discussed results of a significantly moderate correlation between ozone variability and the death rate, the excess mortality assessed by the epidemiological method described in this section confirms the far-reaching impact in the form of man-made funeral pyre burn feedback. Though a variety of pollutants are released as a result of funeral pyres, the effect of ozone alone was traced and investigated in the present study due to limitations in background data and further complexities. Our observation warrants additional health precautions and mitigation strategies to reduce the concentrations of the toxic gas (surface ozone) being produced as a secondary pollutant from anthropogenic activities such as vehicular motion and pyre burning. However, the authors do caution the readers that other factors, such as reductions in particulate matter, could also have contributed to the enhancement in O_3_ via the reduced extinction of solar radiation, which enhances the actinic flux [19,20]. An increase in ozone is mainly attributed to an increase in the ambient concentration of NO_2_ and solar flux. A decline in particulate matter in the atmosphere increases the solar flux, which can escalate the local production of ozone and transport. This makes ozone a leading air pollutant that affects air quality. PM_2.5_ is another potential air pollutant that has a detrimental role in changing the air quality in Delhi, but the mandatory use of face masks and face shields might have screened it from entering the lungs.

## 4. Summary and Conclusions

The funeral rites of those who succumb to COVID-19 are strictly guided by world-wide protocols, and religions have not much influence despite elaborately laid-out rituals. While there is no solid evidence, so far, suggesting that the smoke generated from a funeral pyre can cause COVID-19 directly, nor is there any risk in collecting the ashes for the final rites, the present study points to an indirect feedback mechanism whereby the unscientific and massive burning of pyres can aggravate the COVID-19 crisis and mortality through a degradation in air quality. It highlights the role of degraded air quality, with enhanced levels of toxic gases such as surface ozone and other pollutants, in rendering susceptible patients more vulnerable to the ill effects of COVID-19. It was noticed that the mean AQI during the peak phase of the second wave of COVID-19 was approximately 175 for various stations in New Delhi, with the AQI rising above 300 for several days. The authors highlight the role played by massive open-air cremation in enhancing the pollution level and incrementing the mortality rate (1.27 death per 100,000 population) through epidemiological and statistical analyses and bridging scientific pathways.

Emphasizing the sheer scale of human loss from the disease and the difficulties that it created in managing the hospitalization followed by massive burial, the present study warrants the vital attention of the authorities in carefully handling this grave situation by minimizing the aggravating factors of the pandemic. Nevertheless, the authors highlight the following limitations of the present study. (a) The correlation analysis reported in this study is the simultaneous correlation coefficient observed between ozone exposure and mortality. It is observed that the correlation coefficient decreases as the lag between ozone exposure and mortality increases. It could be due to the fact that the correlation is simply a tool and may not exactly reproduce the cause–effect relation when there are multiple factors controlling the mortality. Moreover, the death rate is dependent on the medical facilities available from time to time. Hence, this analysis alone is not sufficient to identify the lag, and we report the simultaneous correlation only. (b) COVID-19 affects the number of funeral pyres and enhanced surface ozone as the number of deaths increases. While not denying this fact, rather, the authors are curious about the opposite effect, which involves a positive feedback: the rise in mortality could lead to more pyre burning and hence to the deterioration of air quality through the release of toxic gases and thereby could lead to enhanced mortality. (c) The impact of the emergence of a new variant of SARS-CoV-2 on mortality needs to be investigated. While this aspect is beyond the scope of the present work, the authors strongly believe that a fraction of the enhanced mortality can be attributed to the deteriorated air quality, as evident from the analysis using AirQ+. This is further confirmed by the presence of increased levels of surface ozone using satellite measurements. Additional analysis using satellite-derived tropospheric ozone (from AURA OMI data) reveals enhanced surface ozone during the lockdown in 2021.

Keeping in mind that the next wave of COVID-19 could lead to further fatalities, it is essential to have proper measures in place both to contain the spread of the disease and to alleviate the air pollution arising from unscientific disposal methods. The authors propose to adopt modern technologies in scientifically disposing of the bodies of the victims, such as via electric and gas crematoriums or burial in deep graves, and to strictly adhere to the World Health Organization’s (WHO) guidelines and protocols in this regard.

## Data Availability

The air quality data over Delhi were accessed from the publicly available repository of the Central Pollution Control Board under the Ministry of Environment, Forests and Climate Change, Government of India: https://app.cpcbccr.com/AQI_India, accessed on 12 May 2022.

## Supplementary Materials

The following supporting information can be downloaded at: https://www.mdpi.com/article/10.3390/toxics10060306/s1, Figure S1: Reports of pyre burning ill effects: Local newspaper dated 1 May 2021 describing the situation of residents unable to tolerate the smoke being emitted from the pyres in another Indian city, Hyderabad. Figure S2: Daily variation of the tropospheric column ozone data (AURA-OMI) during the lock-down periods in 2020 and 2021. (The tropospheric ozone is calculated by subtracting stratospheric column ozone from the total column ozone data).
