# Supplementary material for: Exacerbation of Fatality Rates Induced by Poor Air Quality Due to Open-Air Mass Funeral Pyre Cremation during the Second Wave of COVID-19
Source: Toxics. 2022 Jun 6;10(6):306. doi: 10.3390/toxics10060306 (PMC9227097; doi:10.3390/toxics10060306)
Supplement: Supplementary file 1 [file toxics-10-00306-s001.zip › toxics-1747839-supplementary/figS/Figure S1.pdf]

HYDERABAD

As COVID bodies burn, residents fume

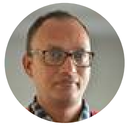

Serish Nanisetti

HYDERABAD, MAY 01, 2021 23:41 IST
UPDATED: MAY 01, 2021 23:43 IST

SHARE ARTICLE PRINT A A A

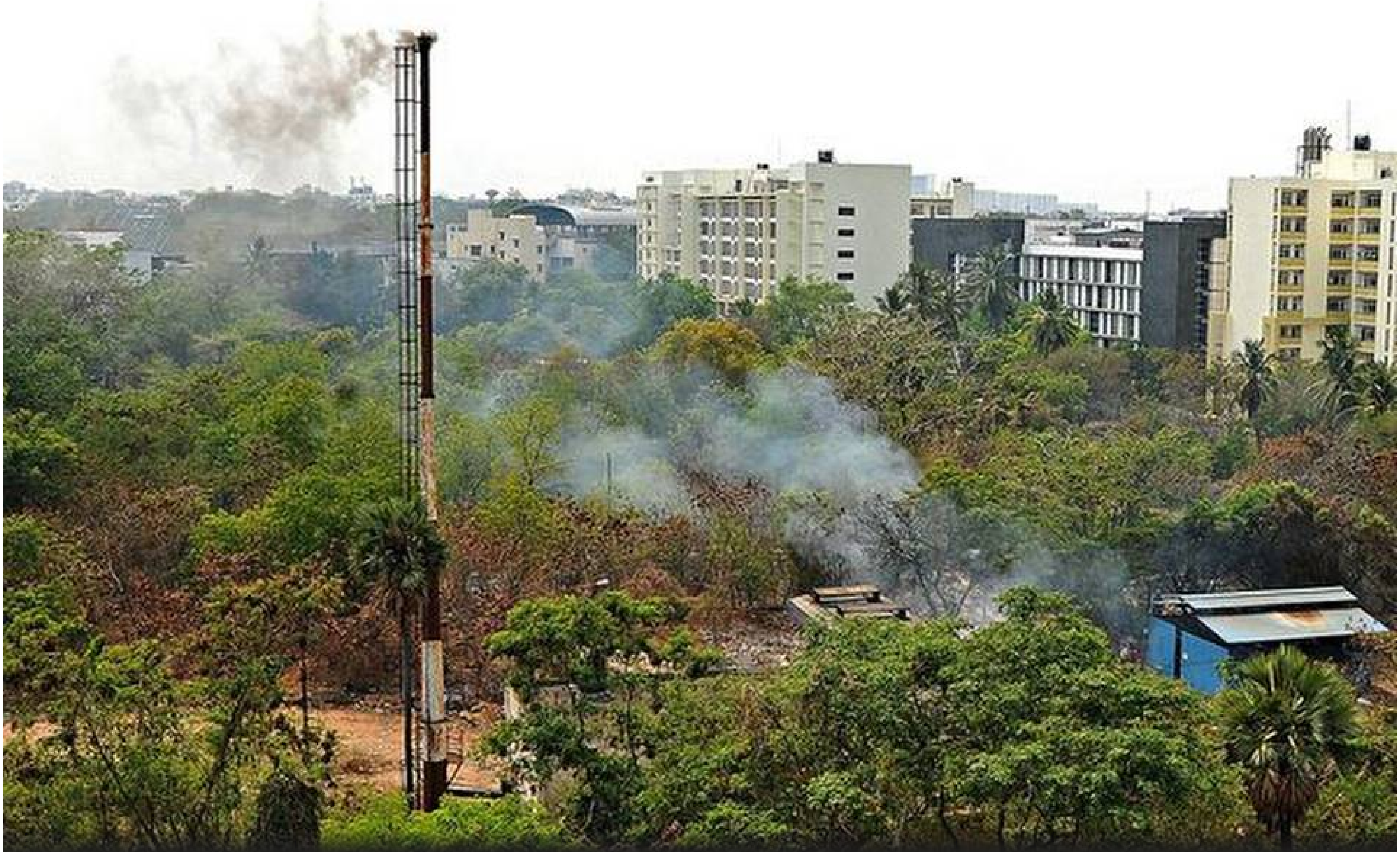

Mass cremation of COVID victims at ESI crematorium near SR Nagar in Hyderabad.

People living near crematoriums forced to lock themselves up, trees turn brown

“Now he will pour diesel on the pyre to make it burn quickly,” says G. Ramulu watching from the terrace of his three-storeyed house in Bapu Nagar. And just as he predicts, a crematorium worker runs with a jerrycan of fuel and sloshes it over the wood pile making the fire roar into the air.

Four other pyres burn simultaneously, and two more bodies arrive in ambulances in quick succession at noon time on Saturday. The trees in the crematorium have turned brown by the continuous burning of corpses of COVID patients.

As the number of cremations go up in the city, residents living in the surrounding areas are being forced to lock themselves in their homes. While Hyderabad has two cremation grounds on either bank of the Musi River, one near Purana Pul and the other at Amberpet, a large number of cremations are taking place in the grounds which are within the city. The Panjagutta cremation ground is almost in the heart of the city surrounded by Banjara Hills and Ameerpet. The ESI Cremation ground is near the densely populated Sanjeeva Reddy Nagar.

“We are able to stand here because the wind is blowing this way. If it blows the other way, we cannot even stand here. The smell of burning flesh is overpowering. In the evening, they burn a greater number of bodies, and it becomes worse,” says G. Goverdhan, a bank employee who lives in Bapu Nagar. The crematorium is fringed by a number of apartment complexes whose residents have raised an alarm.

“People are bringing coronavirus affected corpses into the ground and burning them. The resulting acrid some is become unbearable not only to us and also to other apartment blocks and Bapu Nagar basti. It may be a serious health hazard,” a resident of Rajasree Residency complained to the commissioner of Greater Hyderabad Municipal Corporation.

“The bodies are being burnt 24x7, it is not just bad smell. Small droplets are settling down on clothes, vessels and anything kept outside. The situation has become worse over the 20 days,” says M.A. Fayazuddin of Devarkonda Basti, which abuts the Panjagutta crematorium.

“The GHMC should find a site that is away from the city to burn so many bodies,” says Mr. Fayazuddin.

There are reports of residents living near Amberpet crematorium leaving for their native places unable to bear the smoke being belched out from the burning bodies. But for citizens living in makeshift houses on either side of the Musi River, there is no escaping the smoke and smell of burning bodies emanating from the 100-feet tall chimney stacks.

Printable version | Oct 17, 2021 10:45:56 PM | https://www.thehindu.com/news/cities/Hyderabad/as-covid-bodies-burn-residents-fume/article34460264.ece

© THG PUBLISHING PVT LTD.
