# Supplementary material for: Exacerbation of Fatality Rates Induced by Poor Air Quality Due to Open-Air Mass Funeral Pyre Cremation during the Second Wave of COVID-19
Source: Toxics. 2022 Jun 6;10(6):306. doi: 10.3390/toxics10060306 (PMC9227097; doi:10.3390/toxics10060306)
Supplement: Supplementary file 1 [file toxics-10-00306-s001.zip › toxics-1747839-supplementary/figS/Figure S2.pdf]

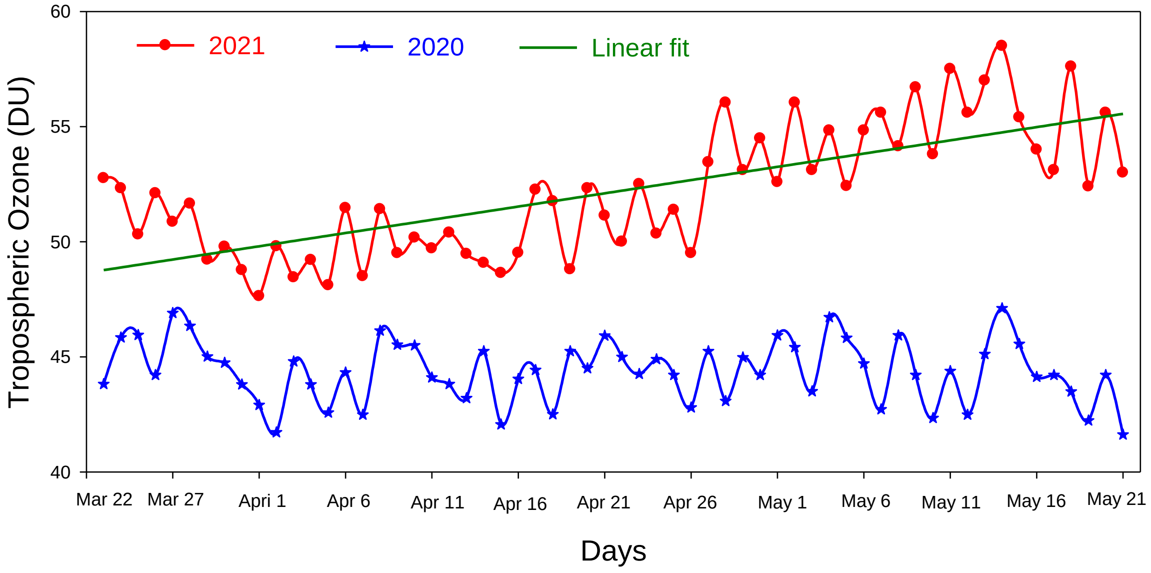

Figure S2. Daily variation of the tropospheric column ozone data (AURA-OMI) during the lock-down periods in 2020 and 2021. (The tropospheric ozone is calculated by subtracting stratospheric column ozone from the total column ozone data).
